# Supplementary material for: The Most Demanding Exercise in Different Training Tasks in Professional Female Futsal: A Mid-Season Study through Principal Component Analysis
Source: Healthcare (Basel). 2022 May 2;10(5):838. doi: 10.3390/healthcare10050838 (PMC9140358; doi:10.3390/healthcare10050838)
Supplement: Supplementary file 1 [file healthcare-10-00838-s001.zip › healthcare-1673771-supplementary.pdf]

Table S1. Descriptive statistics of the training load variables.

Variables	Values							
	N Data		Mean	Median	Stand. Deviat.	Percentile		
	Valid	Loss				25	50	75
**Cluster 1 (preventive exercises)**								
HR % (50-60)	212	129	23.76	18.66	21.78	3.54	18.66	39.38
HR % (70-80)	212	129	22.81	21.17	20.86	2.53	21.17	38.06
HR % (90-95)	212	129	1.28	0.00	5.43	0.00	0.00	0.00
MAX Acc (m/s^2)	212	129	4.35	4.33	2.03	3.10	4.33	5.58
Acc Abs (2-3)/min	212	129	1.30	1.07	1.15	0.34	1.07	2.01
Acc Abs (4-5)/min	212	129	0.12	0.04	0.18	0.00	0.04	0.16
Acc Abs (5-6)/min	212	129	0.04	0.00	0.08	0.00	0.00	0.07
Dec Abs (-6. -5)/min	212	129	0.03	0.00	0.07	0.00	0.00	0.00
Impacts (0-3) G	212	129	1121.28	815.50	940.36	431.50	815.50	1565.75
Impacts (3-5)/min	212	129	5.45	2.70	8.14	0.33	2.70	9.43
Impacts (5-8)/min	212	129	6.97	0.81	18.12	0.04	0.81	3.23
Impacts (8-100)/min	212	129	1.89	0.03	5.80	0.00	0.03	0.38
Landing (5-8)/min	212	129	0.18	0.04	0.40	0.00	0.04	0.23
**Cluster 2 (analytical situations)**								
HR % (50-60)	321	9	16.96	14.12	16.27	2.05	14.12	26.82
HR % (80-90)	321	9	16.18	7.94	19.37	0.00	7.94	28.49
DSL/min	321	9	0.82	0.47	0.95	0.18	0.47	1.11
Vel Abs (0-6) (m·min^{-1})	321	9	25.00	25.05	7.85	19.96	25.05	30.45
Acc/min	321	9	27.48	27.50	6.90	23.07	27.50	31.67
Acc Abs (1-2)/min	321	9	6.56	6.33	2.91	4.34	6.33	8.38
Acc Abs (2-3)/min	321	9	2.37	2.17	1.32	1.48	2.17	3.07
Acc Abs (4-5)/min	321	9	0.15	0.06	0.22	0.00	0.06	0.21
Dec Abs (-5. -4)/min	321	9	0.16	0.09	0.21	0.00	0.09	0.26
Impacts (0-3) G	321	9	1601.88	1313.00	1328.51	702.50	1313.00	2044.50
Impacts (5-8) G	321	9	31.52	20.00	35.02	8.00	20.00	43.50
Impacts (0-3) min	321	9	105.10	110.43	55.60	74.48	110.43	139.43
Impacts (5-8)/min	321	9	7.33	1.86	23.81	0.84	1.86	3.42
**Cluster 3 (exercises in mid court)**								
Expl dist (m)	188	4	9.18	9.18	5.37	5.03	9.18	12.55
HR % (50-60)	191	1	10.88	2.60	17.15	0.00	2.60	14.36
HR % (80-90)	191	1	26.57	24.50	21.26	8.35	24.50	41.99
HR % (90-95)	191	1	19.15	6.31	22.77	0.00	6.31	36.67
HR % (>95)	191	1	4.71	0.00	11.24	0.00	0.00	3.13
Vel Abs (18-21) (m·min^{-1})	191	1	1.41	0.00	3.67	0.00	0.00	0.88
Acc/min	191	1	26.86	27.49	7.68	24.80	27.49	30.59
Dist Acc	172	20	4.57	3.37	5.87	1.56	3.37	5.87
MAX Acc (m/s^2)	191	1	4.72	4.59	1.62	3.88	4.59	5.33
Acc Abs (3-4)/min	191	1	0.97	0.86	0.74	0.45	0.86	1.31
Acc Abs (5-6)/min	191	1	0.06	0.00	0.14	0.00	0.00	0.04

Acc Abs (6-10)/min	191	1	0.04	0.00	0.13	0.00	0.00	0.00
Dec Abs (-2. -1)/min	191	1	6.97	6.96	2.79	5.37	6.96	8.42
Impacts (0-3) G	191	1	1424.01	1043.00	1897.06	586.00	1043.00	1457.00
Impacts (3-5) G	191	1	135.97	96.00	183.35	47.00	96.00	155.00
Impacts (0-3) min	191	1	113.70	123.55	65.02	65.83	123.55	159.45
Impacts (5-8)/min	191	1	17.53	3.40	44.20	1.32	3.40	5.62
**Cluster 4 (exercises in ¾ of the court)**								
Expl dist (m)	492	102	9.88	10.41	5.15	6.58	10.41	13.38
HSR Abs (m·min^{-1})	495	99	0.69	0.00	2.15	0.00	0.00	0.00
HR % (50-60)	495	99	10.42	2.54	15.63	0.00	2.54	16.96
HR % (70-80)	495	99	17.68	14.51	16.05	5.76	14.51	24.55
HR % (80-90)	495	99	27.40	26.57	20.01	11.61	26.57	40.12
Acc/min	495	99	25.80	26.80	7.86	23.90	26.85	29.44
Acc Abs (2-3)/min	495	99	2.76	2.78	1.36	1.99	2.78	3.47
Acc Abs (3-4)/min	495	99	0.95	0.86	0.74	0.45	0.86	1.31
Acc Abs (4-5)/min	495	99	0.25	0.20	0.33	0.00	0.20	0.34
Acc Abs (6-10)/min	495	99	0.04	0.00	0.13	0.00	0.00	0.00
Dec Abs (-1, 0)/min	495	99	15.31	15.50	5.40	13.27	15.50	18.20
Impacts (3-5) G	495	99	134.37	111.00	108.93	60.00	111.00	185.00
Impacts (5-8)/min	495	99	17.67	4.13	42.13	2.25	4.13	6.88
Player Load /min	495	99	1.02	1.06	0.42	0.77	1.06	1.34
Power Metabolic (kg·s)	495	99	199.73	212.30	83.30	160.04	212.30	258.34
**Cluster 5 (exercises in full court)**								
Expl dist (m)	590	4	9.98	10.92	5.24	7.01	10.92	13.57
HML (10-25.5) (m)	593	1	111.31	104.46	78.91	42.78	104.46	163.83
HML (25.5-35) (m	593	1	80.18	75.43	62.52	25.19	75.43	120.96
Vel Abs (18-21) (m·min^{-1})	593	1	2.01	1.07	2.80	0.00	1.07	2.72
Acc/min	593	1	25.64	26.81	8.44	24.45	26.81	28.84
Acc Abs (5-6)/min	593	1	0.21	0.14	0.29	0.00	0.14	0.28
Acc Abs (6-10)/min	593	1	0.04	0.00	0.11	0.00	0.00	0.07
Dec Abs (-2, -1)/min	593	1	6.20	6.35	2.60	5.20	6.35	7.36
Dec Abs (-6, -5)/min	593	1	0.07	0.00	0.14	0.00	0.00	0.11
Dec Abs (-10, -6)/min	593	1	0.03	0.00	0.10	0.00	0.00	0.00
Impacts (3-5) G	593	1	151.42	146.00	97.75	81.00	146.00	220.00
Impacts (5-8)/min	593	1	11.24	4.34	30.01	2.43	4.34	6.40
**Cluster 6 (superiorities/inferiorities)**								
Expl dist (m)	200	394	9.91	10.21	4.99	5.94	10.21	13.08
Dist (m·min^{-1})	103	391	69.27	69.68	29.69	46.59	69.68	94.80
HR % (50-60)	203	391	9.60	2.88	16.59	0.00	2.88	10.14
HR % (70-80)	203	391	20.91	19.18	17.60	6.71	19.18	28.85
MAX HR (bpm)	203	391	169.70	175.00	20.91	164.00	175.00	183.00
Rel HR %	203	391	77.72	79.72	79.10	10.86	72.30	79.10
Vel Abs (18-21) (m·min^{-1})	203	391	2.67	0.45	5.22	0.00	0.45	2.15
Acc/min	203	391	27.51	28.01	6.41	25.10	28.01	31.25

Dist Acc	181	413	5.28	4.02	8.58	1.64	4.02	6.55
MAX Acc ($m \cdot s^2$)	203	391	4.71	4.32	1.65	3.75	4.32	5.28
Acc Abs (0-1)/min	203	391	17.09	17.51	4.59	15.08	17.51	20.03
Acc Abs (2-3)/min	203	391	2.69	2.68	1.28	1.90	2.68	3.39
Acc Abs (4-5)/min	203	391	0.24	0.15	0.32	0.00	0.15	0.43
Dec Abs (-4, -3)/min	203	391	1.00	0.88	0.67	0.53	0.88	1.31
Dec Abs (-5, -4)/min	203	391	0.31	0.24	0.30	0.00	0.24	0.46
Impacts (0-3) min	203	391	104.74	109.22	67.96	61.76	109.22	158.55
